# Immunotherapy in endometrial cancer: mechanisms, clinical evidence, and future directions

**DOI:** 10.3389/fimmu.2025.1697065

**Published:** 2026-01-09

**Authors:** Mengyi Lian, Chengwei Zhang, Tianye Li, Aiming Wang

**Affiliations:** 1 Department of Obstetrics and Gynecology, Longquan People’s Hospital, Lishui, China; 2 Clinical Medical College, Inner Mongolia University for Nationalities, Tongliao, Inner Mongolia Autonomous Region, China; 3 Innovative Regenerative Medicine, Graduate School of Medicine, Kansai Medical University, Hirakata, Osaka, Japan; 4 Department of Gynecology, The Second Affiliated Hospital, Zhejiang University School of Medicine, Zhejiang University, Hangzhou, China; 5 The Sixth Medical Center of the Chinese People’s Liberation Army (PLA) General Hospital, Beijing, China

**Keywords:** endometrial cancer, immunotherapy, immune checkpoint inhibitors, molecular classification, MMR-deficiency, PD-1, pembrolizumab, dostarlimab

## Abstract

Endometrial cancer (EC) treatment has been revolutionized by the integration of immunotherapy, particularly for molecularly defined subsets of patients. The classification of EC into DNA polymerase epsilon-mutated (POLE-mutant), mismatch repair-deficient (dMMR), p53-abnormal, and no specific molecular profile (NSMP) subtypes provides a critical framework for predicting response to immune checkpoint blockade. dMMR and POLE-mutant tumors, with their hypermutated and immunogenic phenotypes, demonstrate exceptional sensitivity to Programmed Death-1(PD-1) inhibitors such as pembrolizumab and dostarlimab in clinical trials. In contrast, overcoming the immunoresistant nature of NSMP and p53-abnormal EC requires innovative combinations, exemplified by the success of pembrolizumab plus the multitargeted tyrosine kinase inhibitor lenvatinib. Recent practice-changing clinical trials have further established combination strategies incorporating PD-1 blockade with chemotherapy as a new first-line standard for advanced disease, marking a paradigm shift in the management of advanced EC. This review synthesizes the mechanistic basis for these approaches, the compelling clinical evidence supporting approved therapies, and the frontier of investigational strategies, including cellular therapies, novel immune checkpoints, and rational combination regimens—aimed at expanding the benefit of immunotherapy to a broader range of patients with EC.

## 1 Introduction

Endometrial cancer (EC) is a common gynecologic malignancy with a rising global incidence. In 2020, an estimated 417,367 new EC cases and 97,370 deaths occurred worldwide (1). Incidence continues to increase, particularly among postmenopausal women—despite declining age-standardized mortality in some regions (2, 3). In advanced and recurrent disease, outcomes remain poor due to limited responsiveness to conventional chemotherapy and hormonal therapy (4–6). The advent of molecular classification established by The Cancer Genome Atlas (TCGA)—including DNA polymerase epsilon-mutated (POLE-mutant), mismatch repair-deficient (dMMR)/microsatellite instability-high (MSI-H), p53-abnormal (copy-number high), and no specific molecular profile (NSMP)—has transformed prognostic stratification and informed therapeutic selection (4–6).

Recent approvals of Programmed Death-1 (PD-1) inhibitors, such as pembrolizumab and dostarlimab, have established immunotherapy in advanced EC. Combination regimens have demonstrated efficacy, extending the potential benefits of immunotherapy to patients with mismatch repair-proficient (pMMR) disease (6). Ongoing phase III trials are evaluating immune checkpoint inhibitors (ICIs) with chemotherapy or targeted agents in first-line settings (7). Preclinical studies are also investigating innate immunity modulators, cytokine therapies, CAR-T cells, tumor-infiltrating lymphocytes (TILs), and therapeutic vaccines aimed at converting immune “cold” tumors into “hot” ones (8). This review integrates mechanistic insights with clinical evidence and highlights future strategies, including novel immune targets, predictive biomarkers, rational combinations, and translational challenges.

## 2 Molecular classification and its impact on immunotherapy in endometrial cancer

**Figure 1:**
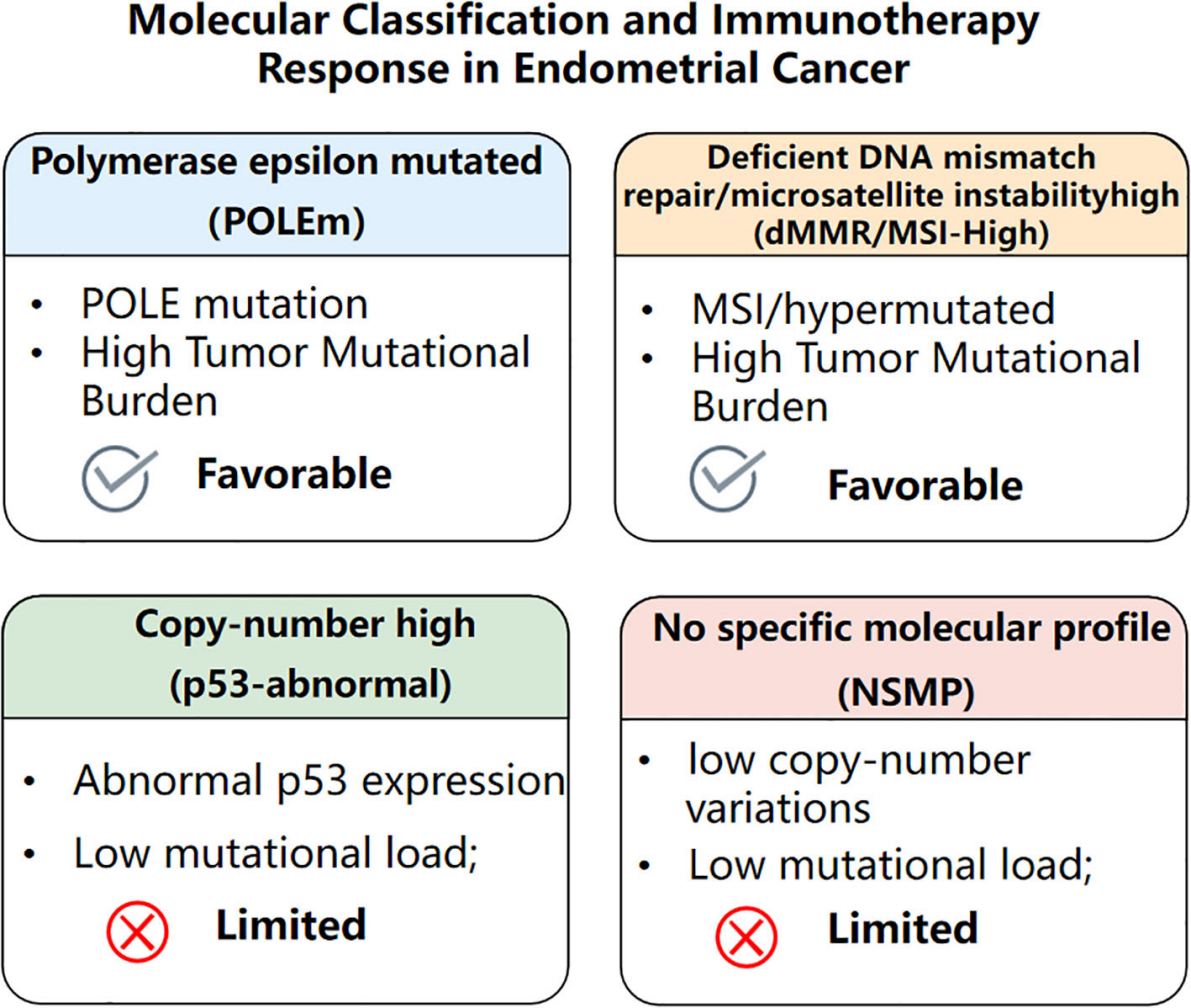
Molecular classification and immunotherapy response in endometrial cancer. The figure summarizes the four molecular subtypes—POLE-mutant, dMMR/MSI-H, p53-abnormal, and no specific molecular profile (NSMP)—along with their key molecular features and predicted responsiveness to immune checkpoint inhibition. POLE-mutated and dMMR/MSI-H subtypes, which exhibit high tumor mutational burden, show favorable responses. In contrast, p53-abnormal and NSMP subtypes, characterized by low mutational burden, are associated with limited benefit from immunotherapy.

The molecular classification of EC, established by The TCGA, has significantly advanced our understanding of tumor biology and response to treatment, particularly immunotherapy (9–11). EC is stratified into four molecular subtypes: POLE-mutant, dMMR/MSI-H, p53-abnormal, and NSMP (11, 12). These subgroups are associated with varying clinical outcomes and responses to therapy (**Figure 1**). For example, POLE-mutant tumors are characterized by an exceptionally high tumor mutational burden (TMB) and strong neoantigenicity, which result in robust T-cell infiltration and immunogenic tumor microenvironments. These tumors demonstrate excellent prognosis and remarkable sensitivity to immune checkpoint blockade (13).

Similarly, dMMR/MSI-H tumors, comprising approximately 25–30% of ECs, show elevated TMB and marked immune activation, including increased CD8^+^ T cell infiltration and PD-L1 expression (14). These features underlie the durable clinical responses observed with PD-1 inhibitors such as pembrolizumab and dostarlimab in this subset (4, 15).

In contrast, p53-abnormal tumors commonly encompass serous or high-grade endometrioid histologies, which exhibit low immune cell infiltration and high expression of immunosuppressive signatures. These tumors are generally resistant to checkpoint inhibitors and may require combinatorial approaches (16). NSMP tumors, which are copy-number low and often harbor CTNNB1 or PTEN mutations, are considered immune “cold” with low PD-L1 expression and minimal immune infiltration generally. Their response to ICIs is limited, although trials are ongoing to explore combinations with anti-angiogenics or epigenetic modifiers (17, 18). Further pan-cancer analyses have highlighted the potential of Runt-related transcription factor (RUNX) family proteins as carcinogenic biomarkers, which may also inform subtype-specific therapeutic targeting in endometrial cancer (19). Taken together, this classification provides a valuable framework for precision immunotherapy in EC, guiding patient selection and tailoring treatment strategies (20).

## 3 Immunotherapy in endometrial cancer: mechanisms and strategies

### 3.1 PD-1/PD-L1 blockade

PD-1 is a cell surface receptor belonging to the immunoglobulin superfamily. Expressed on immune cells—including T cells, NK cells, B lymphocytes, macrophages, dendritic cells, and monocytes—PD-1 modulates immune responses by inhibiting T cell and Treg activation (21, 22). Programmed Death-Ligand 1 (PD-L1) is an immune checkpoint protein expressed on tumor and normal cells that binds to PD-1 on immune cells, suppressing immune responses to promote self-tolerance and enable cancer immune evasion (23). PD-1/PD-L1 inhibitors block this interaction, thereby releasing the brakes on T cell-mediated anti-tumor immunity. This mechanism has proven effective in treating multiple cancers, including EC (24, 25). While offering the potential for durable responses, these agents can also cause immune-related adverse events (irAEs) due to inflammatory side effects (26, 27). The introduction of PD-1 inhibitors has brought meaningful progress to the treatment landscape of endometrial cancer, particularly in tumors with dMMR or MSI-H. These subtypes, known for their elevated tumor mutational burden, are especially responsive to immunotherapy (28). Among the most studied agents, pembrolizumab has been demonstrated robust activity in the dMMR/MSI-H population. Pembrolizumab has received FDA approval for multiple cancer types, including advanced EC (29). In the KEYNOTE-158 trial, pembrolizumab monotherapy achieved an objective response rate (ORR) of 57.1%, with durable responses and a median progression-free survival (PFS) of around 13 months in dMMR/MSI-H EC (4). These results have been confirmed with longer follow-up (30). In contrast, the majority of EC patients have microsatellite-stable (MSS) or mismatch repair-proficient (pMMR) tumors, where response rates to pembrolizumab monotherapy are low (ORR <15%) (4).

Similarly, Dostarlimab, another well-established PD-1 inhibitor, is a humanized monoclonal antibody of the IgG4 isotype that targets the PD-1 receptor (31). Dostarlimab monotherapy, evaluated in the phase I GARNET trial, demonstrated substantial efficacy, particularly in dMMR tumors. Among dMMR patients, it achieved an ORR of 43.5% (15). Building on the single-agent activity, the combination of dostarlimab with carboplatin and paclitaxel chemotherapy was evaluated as first-line treatment in the phase III RUBY/ENGOT-EN6-NSGO/GOG-3031 trial involving 494 patients with advanced or recurrent endometrial cancer. This combination regimen significantly outperformed placebo plus chemotherapy. Most notably, it demonstrated a meaningful improvement in 24-month progression-free survival (PFS) rates both in the overall study population (36.1% vs. 18.1%) and, strikingly, in the dMMR/MSI-H subgroup (61.4% vs. 15.7%), representing a substantial clinical benefit (Hazard Ratio [HR] for progression or death in dMMR was 0.30) (5, 15). These robust results led to the expanded FDA approval of dostarlimab in combination with chemotherapy for primary advanced or recurrent dMMR/MSI-H EC in 2024.

Similarly, the phase III NRG-GY018 trial evaluated pembrolizumab in combination with carboplatin and paclitaxel chemotherapy versus placebo plus chemotherapy as a first-line treatment for advanced or recurrent endometrial cancer. This regimen also demonstrated a significant improvement in PFS across molecular subtypes. The most pronounced benefit was observed in the dMMR/MSI-H population, where pembrolizumab plus chemotherapy significantly reduced the risk of disease progression or death by 66% (HR 0.34) compared to chemotherapy alone, with median PFS not reached versus 8.3 months, respectively (32). Notably, the magnitude of PFS benefit in dMMR tumors was nearly identical to that observed in the RUBY trial, collectively confirming the transformative activity of combining a PD-1 inhibitor with chemotherapy in the first-line setting for this patient subgroup (6, 33).

Beyond PD-1, cytotoxic T-lymphocyte–associated antigen-4 (CTLA-4) also dampens antitumor immunity by delivering inhibitory signals to T cells (34). Zalifrelimab (AGEN1884) is a monoclonal antibody that targets CTLA-4 and counters this checkpoint to enhance immune activation (35). In a phase II, open-label study of balstilimab plus zalifrelimab as second-line therapy for advanced cervical cancer, 10 of 125 patients achieved complete responses and 22 had partial responses (overall response rate 25.6%); 64.2% of responses were ongoing at 12 months (36). The ORR was 32.8% in PD-L1–positive tumors versus 9.1% in PD-L1–negative disease. Together, these findings indicate that CTLA-4 blockade can complement PD-1 inhibition to improve antitumor efficacy.

### 3.2 Combined checkpoint and targeted therapy

While the combination of PD-1 blockade with chemotherapy has become a new standard for first-line treatment, particularly in dMMR/MSI-H patients (as demonstrated by the RUBY and NRG-GY018 trials), overcoming resistance in pMMR tumors remains a challenge (37, 38). This has spurred the investigation of immunotherapy combined with targeted agents (39, 40). The combination of pembrolizumab with lenvatinib, a multi-kinase inhibitor targeting VEGF receptors, has significantly improved outcomes in pMMR EC. In the phase III KEYNOTE-775 trial, this combination demonstrated superior efficacy over chemotherapy alone: median progression-free survival (PFS) was 6.6 months versus 3.8 months (hazard ratio [HR] 0.60), overall survival (OS) was 17.4 months versus 12.0 months (HR 0.68), and ORR was 30.3% versus 15.1% (41). In contrast, the phase III LEAP-001 trial evaluating first-line pembrolizumab plus lenvatinib versus chemotherapy met its non-inferiority endpoint for overall survival but did not demonstrate statistically superior survival outcomes (42). For patients with recurrent EC (post-chemotherapy), pembrolizumab plus lenvatinib maintains superiority over chemotherapy in both PFS and response rates, with particularly clinically meaningful benefits observed in pMMR tumors (43). Toxicity includes hypertension, diarrhea, fatigue, and thyroid dysfunction but was manageable with dose reductions.

In addition, the overexpression of receptor tyrosine kinases such as EphA2 has been associated with poor clinical outcomes. Studies have demonstrated that combining EphA2-targeted therapies with inhibitors of the DNA damage response, such as Wee1 kinase inhibitors, can enhance therapeutic efficacy by inducing apoptosis and reducing tumor cell viability (44). This approach is further supported by evidence that targeting DNA damage repair pathways can activate immune responses, suggesting a synergistic potential when combined with immune checkpoint blockade (45). Additionally, the combination of EphA2 inhibitors with histone deacetylase inhibitors has been shown to downregulate survival pathways and reduce tumor burden, highlighting the promise of multitargeted approaches in endometrial cancer treatment (18, 46).

### 3.3 Cellular therapies

Emerging cellular immunotherapies are under active study in endometrial cancer and may offer options for advanced or treatment-resistant disease. Early-phase trials are assessing chimeric antigen receptor T (CAR-T) cells against antigens such as mesothelin, MUC16 (CA-125), Human Epidermal Growth Factor Receptor 2 (HER2), and folate receptor-α (FRα) (NCT03916679, NCT03585764), while preclinical studies highlight Müllerian inhibiting substance type II receptor MISIIR/AMHR2 as EC-relevant targets (47), with MISIIR-directed CAR-T cells showing selective cytotoxicity, patient-derived tumor killing, and tumor growth inhibition in xenograft models with minimal off-target effects. Other approaches under investigation include CAR-macrophages (NCT04660929), tumor-infiltrating lymphocyte therapy in MMRd and POLE-mutant tumors (NCT06481592), NK cell therapies, and TCR-engineered T cells (33, 48, 49). Despite challenges such as antigen heterogeneity, antigen loss, and an immunosuppressive microenvironment, strategies including multi-target or armored CAR designs, intraperitoneal delivery, and combinations with checkpoint blockade are being explored, with ongoing studies expected to define their clinical role.

## 4 Clinical evidence and approved therapies

The compelling clinical efficacy of immune checkpoint inhibitors has led to their regulatory approval and integration into standard treatment paradigms for advanced endometrial cancer, both as monotherapy and in combination regimens. **Table 1** summarizes the key registration trials supporting the approval of these agents. Pembrolizumab and dostarlimab have established roles in dMMR/MSI-H disease, while the combination of pembrolizumab and lenvatinib represents a standard option for pMMR tumors.

**Table 1:** Approved immunotherapy agents for endometrial cancer.

Regimen	Approval year	Biomarker status	Key trial (Phase)	NCT number
Pembrolizumab	2022	MSI-H/dMMR	KEYNOTE-158 (Phase 2)	NCT02628067
Pembrolizumab + Carboplatin + Paclitaxel	2024	All comers	NRG-GY018/KEYNOTE-868 (Phase 3)	NCT03914612
Dostarlimab	2021	MSI-H/dMMR	GARNET (Phase 1/2)	NCT02715284
Dostarlimab + Carboplatin + Paclitaxel	2024	MSI-H/dMMR	RUBY (Phase 3)	NCT03981796
Pembrolizumab + Lenvatinib	2021	pMMR/MSS	KEYNOTE-775 (Phase 3)	NCT03517449
Durvalumab + Carboplatin + Paclitaxel + Olaparib	Under Review	All comers (best in dMMR)	DUO-E (Phase 3)	NCT04269200

MSI-H, Microsatellite instability-high; dMMR, Mismatch repair deficient; pMMR, Mismatch repair-proficient; MSS, Microsatellite-stable.

Additionally, the phase III DUO-E trial further expands the first-line arsenal by evaluating a novel sequential combination strategy. This study investigated durvalumab added to platinum-based chemotherapy, followed by maintenance durvalumab with or without the PARP inhibitor olaparib in newly diagnosed advanced or recurrent EC. The regimen demonstrated a significant improvement in PFS compared to chemotherapy alone. Of particular importance, the greatest benefit was observed in the dMMR/MSI-H subgroup, where the combination of durvalumab and olaparib in the maintenance phase led to a substantial reduction in the risk of disease progression or death (HR: 0.55). This suggests a potential synergistic effect between PARP inhibition and immunotherapy in this molecular subset, providing a compelling rationale for this triple-combination approach and positioning it as a promising new therapeutic option (37).

Beyond the currently approved agents, a number of new immunotherapy strategies are under active clinical investigation (**Table 2**). These include dual checkpoint blockade (such as durvalumab with tremelimumab), combinations of PD-1 inhibitors with chemotherapy, PARP inhibitors, or other targeted agents, as well as antibody–drug conjugates like Sacituzumab, and, notably, trastuzumab deruxtecan (T-DXd), which has demonstrated promising activity in HER2-expressing EC with ORR of 57.5% in the phase II DESTINY-PanTumor02 trial (50–52). Novel approaches such as therapeutic vaccines, oncolytic viruses, and adoptive cell therapies (including TILs) are also being explored.

**Table 2:** Ongoing clinical trials in endometrial cancer immunotherapy.

ClinicalTrials.gov identifier	Regimen	Biomarker status	Phase	Study status
NCT06917092	QL1706 + chemotherapy + Bevacizumab	Unknown	Phase 2	Recruiting
NCT06974110	MOMA-341 + Irinotecan + Immunotherapy	MSI-H/dMMR	Phase 1	Recruiting
NCT06253494	AdHER2DC vaccine +Pembrolizumab + Lenvatinib + N-803	Unknown	Phase 1/2	Recruiting
NCT06132958	Sacituzumab tirumotecan	Unknown	Phase 3	Recruiting
NCT03932409	Pembrolizumab + Paclitaxel + Carboplatin	Unknown	Phase 1	Active, not recruiting
NCT06680739	Durvalumab + Tremelimumab	MSI-H/dMMR	Phase 2	Recruiting
NCT03835819	Pembrolizumab + Mirvetuximab soravtansine	MSS	Phase 2	Active, not recruiting
NCT06989112	Trastuzumab Deruxtecan + Rilvegostomig + Pembrolizumab	pMMR	Phase 3	Recruiting
NCT04486352	Atezolizumab + Talazoparib	Unknown	Phase 1/2	Recruiting
NCT04065269	ATR inhibitor drug ceralasertib (AZD6738) + Olaparib + Durvalumab (MEDI4736)	Unknown	Phase 2	Recruiting
NCT06486441	Sacituzumab Govitecan	Unknown	Phase 3	Recruiting
NCT05819892	Chemotherapy + Dostarlimab	MSI-H/dMMR	Phase 1	Recruiting
NCT06481592	Lifileucel + Cyclophosphamide + Fludarabine + IL-2	Unknown	Phase 2	Recruiting
NCT04652076	Humanized Monoclonal Antibody Targeting Netrin-1 (NP137) + Carboplatin + Paclitaxel + Pembrolizumab	Unknown	Phase 1/2	Active, not recruiting
NCT06518564	Human IgG1 antibody (Avelumab) + ATR inhibitor (M1774)	Unknown	Phase 2	Recruiting
NCT06532539	Cadonilimab + Chemoradiation	Unknown	Phase 2	Recruiting
NCT05812677	Oncolytic Virus Injection (R130)	Unknown	Phase 1	Recruiting
NCT03914612	Pembrolizumab (MK-3475) + paclitaxel + carboplatin	All comers	Phase 3	Active, not recruiting
NCT01174121	Tumor Infiltrating Lymphocytes	Unknown	Phase 2	Recruiting
NCT06278857	Dostarlimab	dMMR	Phase 2	Recruiting
NCT06333314	Dostarlimab	MSI-H/dMMR	Phase 2	Recruiting
NCT04214067	Radiation + pembrolizumab (MK-3475)	dMMR	Phase 3	Active, not recruiting
NCT05086692	Recombinant interleukin-2 (MDNA11) + Pembrolizumab	Unknown	Phase 1/2	Recruiting
NCT03860272	Anti-CTLA-4 human monoclonal antibody (botensilimab)	Unknown	Phase 1	Active, not recruiting
NCT02715284	Dostarlimab	All comers	Phase 1	Recruiting
NCT04660929	Anti-HER2 CAR macrophages (CT-0508) + Pembrolizumab	Unknown	Phase 1	Active, not recruiting
NCT05592626	Bifunctional Antibody-fusion Molecule (STAR0602)	Unknown	Phase 1/2	Recruiting
NCT04034927	Olaparib	Unknown	Phase 2	Active, not recruiting
NCT05572684	NC410 + Pembrolizumab	MSS/MSI-L	Phase 1/2	Active, not recruiting
NCT05194735	Neoantigen-specific TCR-T cells + Aldesleukin (IL-2)	Unknown	Phase 1/2	Active, not recruiting

PD-1, Programmed Death-1; PD-L1, Programmed Death-Ligand 1; Anti-CTLA-4, Anti-cytotoxic T-lymphocyte antigen 4; HER2, human epidermal growth factor receptor 2; CAR, chimeric antigen receptor; IL-2, Interleukin-2; ATR, Ataxia Telangiectasia and Rad3-Related Protein. MSI-H, Microsatellite instability-high; MSI-L, Microsatellite instability-Low; dMMR, Mismatch repair deficient; pMMR, Mismatch repair-proficient; MSS, Microsatellite-stable.

Most of these studies are in early phases and are designed to establish safety and preliminary activity, but several phase III trials are already underway. Together, these efforts illustrate a shift toward broader and more tailored immunotherapy options for endometrial cancer, with the expectation that upcoming results may further expand treatment opportunities.

## 5 Translational advances and future directions in immunotherapy for endometrial cancer

**Figure 2:**
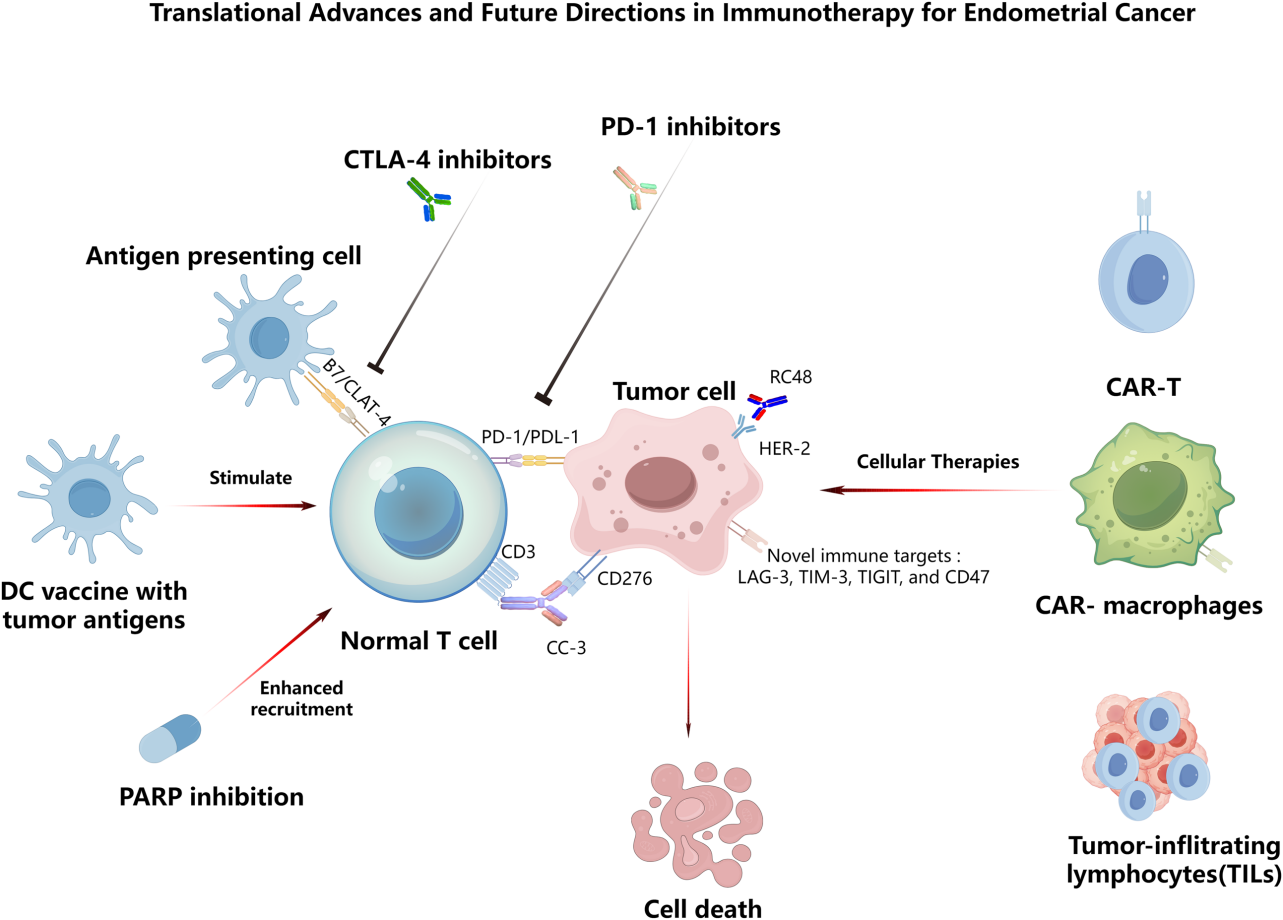
Translational advances and future directions in immunotherapy for endometrial cancer. The schematic summarizes current and emerging strategies: (1) dendritic-cell vaccines presenting tumor antigens to prime endogenous T cells; (2) checkpoint blockade with anti–CTLA-4 and anti–PD-1/PD-L1 to relieve inhibitory signaling; (3) PARP inhibition activating cGAS–STING signaling and chemokine production to recruit CD8^+^ T cells; (4) a B7-H3 (CD276)×CD3 bispecific antibody that bridges tumor cells and T cells to trigger cytotoxicity; (5) the HER2-directed antibody–drug conjugate disitamab vedotin (RC48) for HER2-positive EC; and (6) cellular therapies—including CAR-T cells, CAR-macrophages, and tumor-infiltrating lymphocytes (TILs). Additional emerging targets (LAG-3, TIM-3, TIGIT) and the CD47 are highlighted. Collectively, these approaches aim to enhance antitumor immunity and promote tumor-cell clearance. Abbreviations: DC, dendritic cell; ADC, antibody–drug conjugate; EC, endometrial cancer.

Research on immunotherapy for EC is moving from mechanistic insights to clinical application. In recent years, growing evidence has elucidated the intrinsic immune evasion characteristics of EC, providing a foundation for novel immunotherapeutic strategies. Below, we move from mechanism to tactics and outline what should come next (**Figure 2**).

### 5.1 From immunotherapy resistance mechanisms to targeted interventions

**Figure 3:**
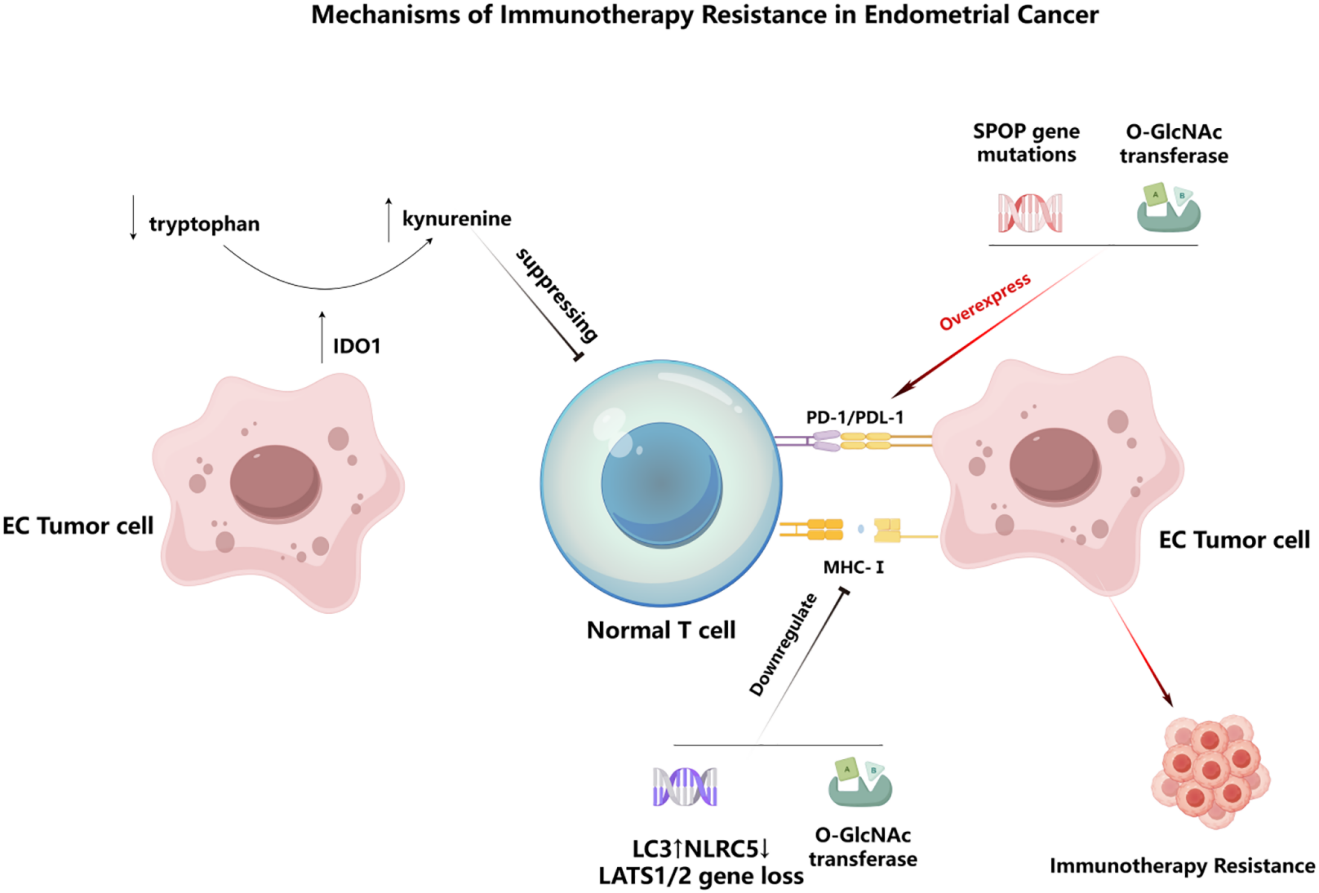
Mechanisms of immunotherapy resistance in endometrial cancer. Loss of LATS1/2 or LC3-mediated selective autophagic degradation of NLRC5 reduces MHC-I expression and impairs antigen presentation, limiting T-cell recognition. SPOP mutations promote PD-L1 overexpression, while O-GlcNAcylation driven by O-GlcNAc transferase (OGT) upregulates PD-L1 and further suppresses MHC-I. In parallel, activation of IDO1 converts tryptophan into kynurenine, which suppresses effector T-cell function. Together, these converging alterations create a profoundly immune-evasive tumor microenvironment and lead to resistance to immune checkpoint blockade.

Recent findings further highlight that both primary and acquired resistance to immunotherapy in endometrial cancer arise from multifactorial defects in tumor–immune interactions. A central barrier is impaired antigen presentation resulting from disruption of the MHC-I machinery. Beyond the loss of LATS1/2 (key regulators of the Hippo signaling pathway) —which suppresses MHC-I transcription—endometrial tumors can also undergo LC3-mediated selective autophagic degradation of NLRC5, the master transcriptional activator of MHC-I genes, thereby further diminishing tumor visibility to cytotoxic T cells (53, 54). At the same time, endometrial tumors frequently activate parallel immunosuppressive pathways. For example, SPOP (a component of the E3 ubiquitin ligase complex) mutations sustain PD-L1 overexpression, while activation of the indolamine 2,3-dioxygenase 1 (IDO1)–kynurenine axis suppresses effector T-cell function and promotes a metabolically hostile microenvironment (54–56). In addition, post-translational modifications such as O-linked N-acetylglucosamine (O-GlcNAc) of the glucocorticoid receptor by O-GlcNAc transferase (OGT) have been shown to simultaneously upregulate PD-L1 and reduce MHC-I, reinforcing a strongly immune-evasive phenotype (57). To provide an integrated overview of these mechanisms, the key pathways driving immune resistance in endometrial cancer are illustrated in **Figure 3**. These converging mechanisms, together with poor T-cell infiltration and enrichment of immunosuppressive stromal components in pMMR and p53-abnormal tumors, help explain the limited effectiveness of immune checkpoint blockade in these subgroups and underscore the need for rational combination strategies. Targeting alternative pathways can substantially reduce tumor immune evasion. For example, in a cohort of 99 patients with type I or II primary endometrial cancer, Brunner et al. reported significantly higher B7-H3 (CD276) expression in advanced tumors than in low-grade tumors (58). Consistent with this, B7-H3 has been implicated as an immunomodulatory molecule within the tumor microenvironment (59). Building on these mechanisms, translational efforts have developed novel interventions: the bispecific antibody CD276×CD3 (CC-3, NCT05999396) simultaneously engages tumor antigens and CD3 to activate T cells (60). Furthermore, given its high and specific expression in tumors, B7-H3 has become an attractive target for antibody-drug conjugates (ADCs), with several such agents currently in early-stage clinical development. Furthermore, given its high and specific expression in tumors, B7-H3 has become an attractive target for ADCs, with several such agents currently in early-stage clinical development.

### 5.2 Cellular therapies and tumor microenvironment modulation

Beyond checkpoint blockade, cell-based therapies offer MHC-independent cytotoxicity. CAR-T cells are being developed for targets such as mesothelin, Mucin 16 (MUC16), human epidermal growth factor receptor 2 (HER2), folate receptor alpha (FRα), and Müllerian inhibiting substance type 2 receptor (MISIIR) (47, 61), with MISIIR-CAR-T showing selective cytotoxicity and significant tumor suppression in xenograft models with minimal off-target effects. CAR-macrophages (NCT04660929) can enhance antigen-specific phagocytosis and remodel the tumor microenvironment (TME), while TIL therapy (NCT06481592) and TCR-engineered approaches expand the range of addressable targets (62). These modalities can be combined with TME-modulating agents or oncolytic viruses to improve tumor infiltration and persistence. Recent evidence also suggests that targeting senescent cells within the TME may enhance immunotherapy efficacy by alleviating immunosuppressive niches (63).

### 5.3 Future directions: combination strategies and precision approaches

Future research will focus on transforming immune-resistant EC into an Immune-responsive disease through rational combinations. Clinically, investigational options for endometrial cancer include polyadenosine diphosphate ribose polymerase (PARP) inhibitors and HER2-targeted antibodies (64). PARP inhibitors are standard for BRCA1/2-mutant or homologous-recombination–deficient (HRD) cancers (65). In p53abn endometrial cancer, 25% of patients show HRD and <5% harbor BRCA1/2 mutations (66, 67). Early studies indicate benefit from combining checkpoint blockade with PARP inhibition in HRD cases (53). Mechanistically, ICIs plus PARP inhibitors such as olaparib, which activates the cGAS/STING pathway in tumor cells, leading to dendritic cell priming and enhanced CD8^+^ T-cell recruitment (54, 55); Moreover, HER2 amplification occurs more frequently in p53abn endometrial cancers than in other molecular subtypes, affecting up to 25% of cases (68). Therefore, the antibody-drug conjugate disitamab vedotin (RC48) combined with the PD-1 inhibitor toripalimab has achieved tumor shrinkage in HER2-positive EC (NCT04280341) (69) Additionally, early studies suggest therapeutic value in EC for combinations such as ICIs with anti-angiogenics to normalize vasculature and reduce MDSCs (41, 70); and ICIs plus TGF-β or IDO inhibitors to reverse immunosuppression (71–73).

Beyond PD-1/PD-L1, novel immune targets such as lymphocyte-activation gene 3 (LAG-3), T cell immunoglobulin and mucin-domain containing-3 (TIM-3), T-cell immunoglobulin and ITIM domain (TIGIT), and CD47 are under active investigation (4, 74). Predictive biomarkers are expanding beyond MMR/MSI and TMB to include Immunoscore, immune gene signatures (e.g., CXCL9/10), and circulating markers such as TCR clonality, IFN signatures, and ctDNA dynamics (75). Emerging technologies are also harnessing small extracellular vesicles as minimally invasive biomarkers for cancer diagnosis and monitoring, offering potential for real-time assessment of immunotherapy response (76). On the personalized therapy front, neoantigen-based vaccines for POLE-mutant or MSI-H tumors, autologous dendritic cell (DC) vaccines—in which DCs are loaded ex vivo with tumor antigens and administered to stimulate specific T- and B-cell responses—as well as TCR-engineered therapies targeting cancer-testis antigens (NY-ESO-1, MAGE-A4), represent key frontiers (77). Early-stage applications are also being explored in adjuvant and neoadjuvant settings, such as in the KEYNOTE-B21 trial (NCT04634877) and in neoadjuvant studies for advanced dMMR EC (78, 79).

Additionally, the combination of immunotherapy with radiotherapy (immuno-radiotherapy) is an emerging exploratory approach. Radiotherapy can induce immunogenic cell death, releasing tumor antigens and potentially converting the local tumor microenvironment into a more immunogenic state, a phenomenon known as the “abscopal effect” (80). This provides a strong rationale for combining it with ICIs to stimulate systemic anti-tumor immunity. Early-phase clinical trials are currently investigating the safety and efficacy of PD-1/PD-L1 inhibitors in conjunction with radiotherapy for advanced or recurrent endometrial cancer (NCT04214067), although mature data are still awaited (81).

Beyond novel immune checkpoints, agents targeting innate immune pathways represent another frontier for overcoming immunotherapy resistance. Preclinical studies across various cancers have demonstrated the potential of stimulator of interferon genes (STING) agonists to reverse an immunosuppressive tumor microenvironment and enhance T-cell priming (82).

## 6 Conclusion

Immunotherapy has redefined treatment paradigms in endometrial cancer, particularly for molecularly defined subgroups such as MSI-H and POLE-mutant tumors. PD-1/PD-L1 inhibitors alone or in combination with anti-angiogenic or DNA-damage response agents have demonstrated durable efficacy. However, immune exclusion, immunosuppressive stromal elements, and low mutational burden remain major barriers in MMR-proficient disease. Translational and preclinical research is uncovering novel immune evasion mechanisms and therapeutic targets. Future success will depend on refined patient selection, biomarker integration, and rational immunotherapy combinations. As our understanding of the tumor-immune interplay evolves, personalized immunotherapy holds the potential to significantly improve survival and quality of life in endometrial cancer patients.
